# Abdominal vessel depiction on virtual triphasic spectral detector CT: initial clinical experience

**DOI:** 10.1007/s00261-021-03001-2

**Published:** 2021-03-14

**Authors:** Simon Lennartz, Kai Roman Laukamp, Yasmeen Tandon, Michelle Jordan, Nils Große Hokamp, David Zopfs, Lenhard Pennig, Markus Obmann, Robert C. Gilkeson, Karin A. Herrmann, Nikhil Ramaiya, Amit Gupta

**Affiliations:** 1 Department of Radiology, University Hospitals Cleveland Medical Center, 11000 Euclid Ave, Cleveland, OH 44106 USA; 2 Department of Radiology, Case Western Reserve University, Cleveland, OH USA; 3 Department of Diagnostic and Interventional Radiology, Faculty of Medicine and University Hospital Cologne, University Cologne, Kerpener Straße, 62, 50937 Cologne, Germany; 4 Else Kröner Forschungskolleg Clonal Evolution in Cancer, University Hospital Cologne, Weyertal 115b, 50931 Cologne, Germany; 5 Department of Radiology, Massachusetts General Hospital, Harvard Medical School, 55 Fruit Street, White 270, Boston, MA 02114 USA; 6 Department of Radiology, Mayo Clinic, 200 First St SW, Rochester, MN 55905 USA; 7 Department of Radiology and Nuclear Medicine, University Hospital Basel, Basel, Switzerland

**Keywords:** Spectral-detector CT, Virtual monoenergetic images, Virtual non-contrast images, Multiphase CT

## Abstract

**Purpose:**

To evaluate vessel assessment in virtual monoenergetic images (VMI_40keV_) and virtual-non-contrast images (VNC) derived from venous phase spectral detector computed tomography (SDCT) acquisitions in comparison to arterial phase and true non-contrast (TNC) images.

**Methods:**

Triphasic abdominal SDCT was performed in 25 patients including TNC, arterial and venous phase. VMI_40keV_ and VNC were reconstructed from the venous phase and compared to conventional arterial-phase images (CI_art_), TNC and conventional venous-phase images (CI_ven_). Vessel contrast and virtual contrast removal were analyzed with region-of-interest-based measurements and in a qualitative assessment.

**Results:**

Quantitative analysis revealed no significant attenuation differences between TNC and VNC in arterial vessels (*p*-range 0.07–0.47) except for the renal artery (*p* = 0.011). For venous vessels, significant differences between TNC and VNC were found for all veins (*p* < 0.001) except the inferior vena cava (*p* = 0.26), yet these differences remained within a 10 HU range in most patients. No significant attenuation differences were found between CI_art_/VMI_40keV_ in arterial vessels (*p*-range 0.06–0.86). Contrast-to-noise ratio provided by VMI_40keV_ and CI_art_ was equivalent for all arterial vessels assessed (*p*-range 0.14–0.91).

Qualitatively, VMI_40keV_ showed similar enhancement of abdominal and pelvic arteries as CI_art_ and VNC were rated comparable to TNC.

**Conclusion:**

Our study suggests that VNC and VMI_40keV_ derived from single venous-phase SDCT offer comparable assessment of major abdominal vessels as provided by routine triphasic examinations, if no dynamic contrast information is required.

## Introduction

Multiphasic scanning with computed tomography (CT) is performed for various clinical indications including suspected aneurysm rupture, aortic dissection [1, 2], assessment of abdominal aortic aneurysm repair [3–5] or preoperative evaluation of living kidney donors [6]. Despite the unquestioned benefit of triphasic CT scanning to assess vasculature, radiation exposure for patients undergoing such examinations is inherently high as compared to single-phase CT examinations.

The standard single-phase CT imaging in venous phase is sufficient in a broad spectrum of clinical indications. For instance, in cancer patients serial follow-up CT scans are often required which are usually acquired in a single venous contrast phase. Although these exams are adequate for tumor staging, incidental findings within the vascular system such as stenosis, thrombosis or calcifications, may be incidentally detected, for which, unenhanced or arterial phase imaging may be needed for complete assessment. In such cases, additional complementary scans often come at a cost of added radiation exposure and repeated contrast media application.

Dual-energy CT (DECT) has been previously reported as a feasible method to provide virtual non-contrast (VNC) images and low-keV virtual monoenergetic images (VMI) as surrogate for true non-contrast images (TNC) and angiographic acquisitions, respectively [7–14]. DECT allows for detection and quantification of iodine, which can be subtracted subsequently from the original image to obtain VNC images. VMI are calculated as balanced combinations of Compton- and Photoelectric-weighted datasets, resembling an image which would result from the acquisition at a specific energy level. In low-keV VMI, iodine attenuation is markedly increased compared to conventional CT image, as the chosen energy level approximates the absorption maximum of iodine at k-edge of 33.2 keV. Considering this effect, one could speculate that VMI from venous phase images may provide image information similar to that with higher iodine concentration or arterial phase imaging. So far, comparison of such low-keV VMI to angiographic images and VNC to TNC images has only been tested separately with a detector-based DECT.

We hypothesized that VNC and VMI at 40 keV (VMI_40keV_) derived from the same venous phase SDCT examination would enable comparable evaluation as provided by additional TNC and angiographic phase image acquisitions. Hence, in this study, we investigate the added value of VNC and VMI at 40 keV derived from the same venous phase spectral detector CT (SDCT) scan with respect to overall image quality and diagnostic assessment.

## Material and methods

The institutional review board approved this single-center, HIPAA-compliant study and waived informed consent based to its retrospective nature. No scan was performed for the purpose of this study only; each study was clinically indicated.

### Patients

Study participants were identified retrospectively by a systematic search in the picture archiving and communication system (PACS) and radiological information system (RIS) using the following inclusion criteria:

1. Age ≥ 18 years,
2. Contrast-enhanced, triphasic, abdominopelvic SDCT scan comprising unenhanced, arterial and venous phases between April 2017 and May 2018.

Only patients with a complete set of images, including conventional unenhanced, arterial, and venous phase images, as well as VNC and VMI_40keV_ images of the venous phase were included, resulting in a total of 25 patients. These patients were imaged for the following clinical indications: evaluation of an abdominal aortic aneurysm repair (*n* = 7), kidney donor evaluation (*n* = 10), and evaluation of an acute abdomen (*n* = 8, i.e., abdominal bleeding, vessel pathology, and bowel ischemia).

### Image acquisition and postprocessing

All scans were performed on a clinical SDCT scanner (IQon, Philips Healthcare, Best, the Netherlands). The following scanning parameters were employed: tube voltage: 120 kVp, tube current modulation activated (DoseRight 3D-DOM, Philips Healtcare), gantry rotation time 0.40 s, pitch 1.02, collimation 64 × 0.625 mm.

Triphasic contrast-enhanced SDCT scans were performed using a body-weight adapted bolus (1.5 ml/kg) of iodinated contrast agent (Optiray 350 mg/ml, Guerbet) injected via a peripheral vein. Angiographic arterial phase scans were started 8–12 s after a threshold of 100 Hounsfield Units (HU) was reached in the upper descending aorta (bolus triggering method); arterial scan delays were adjusted to the particular clinical question. Venous phase acquisitions started following 70–80 s delay after intravenous contrast media injection.

Images were reconstructed in axial plane using a slice thickness of 3 mm. For reconstruction of VMI_40keV_ and VNC images, a spectral reconstruction algorithm was used (Spectral, B, level 3, Philips Healthcare). Conventional venous and arterial phase images as well as TNC images were reconstructed using a hybrid-iterative reconstruction algorithm, which is established in clinical routine (iDose 4, level 3, Philips Healthcare). Quantitative and qualitative equivalence between these two reconstruction algorithms has previously been demonstrated [15]. Window settings for all reconstructions were set at a window level of 50 and a window width of 360 as a standard. Reviewers were allowed to adjust window settings freely during analysis.

CTDIvol (CT Dose Index-Volumetric) was recorded for each scan to evaluate potential radiation dose savings by the proposed virtual triphasic approach as compared to standard multiphasic acquisition.

### Quantitative image analysis

Attenuation (HU) values and standard deviation (SD) were measured using regions-of-interest (ROI) within the abdominal aorta and its major branches including the celiac trunk, superior mesenteric artery (SMA), renal arteries, common, external and internal iliac arteries and common femoral arteries. Similarly, attenuation values in HU and SD were measured within the inferior vena cava, portal vein, renal veins, common iliac veins and the common femoral veins. ROI were placed within the vessels such that the ROI included the entirety of the vessel lumen and sparing the vessel wall or extravascular circumjacent tissue. ROIs were placed at similar locations within the vessels to ensure comparability of mean values and standard deviation of attenuation as well as signal- and contrast-to-noise ratios for TNC, arterial and venous phase images and VNC and VMI_40keV_ across all images.

### Qualitative assessment

Two board-certified radiologists with eight and ten years of experience independently evaluated VNC and VMI_40keV_ derived from venous phase scans in comparison to the standard TNC and arterial phase images, respectively, using a 5-point Likert scale. In particular, removal of contrast media information in VNC as compared to TNC was evaluated for (1) abdominal aorta, (2) aortic branches (celiac trunk, SMA, and renal arteries), (3) pelvic arteries (as listed above) and (4) abdominal pelvic veins. Arterial vessel enhancement in VMI_40keV_ was compared to arterial phase images in the arterial vessels listed in 1–3, accordingly. Readers were not blinded regarding the image reconstructions type. First, at least to some extent CI_art_ and VMI_40keV_ as well as TNC and VNC are distinguishable by their imaging appearance. Further, we aimed to encourage readers to appreciate also subtle differences to the reference standard reconstructions, being CI_art_ and TNC; therefore, readers were always presented with a full image set of one patient at a time.

For the subgroup of patients who received CT due to evaluation of abdominal aortic aneurysm repair (*n* = 7), readers indicated assessability of the graft on 5-Point Likert scale for VNC and VMI_40keV_ as compared to TNC and arterial phase images. Table 1 shows detailed qualitative criteria for all patients and both subgroups. 

For the subgroup of patients who underwent CT as part of kidney donor evaluation (*n* = 10), readers rated qualitative assessability of arterial [and venous] vessel anatomy of the kidneys comparing VMI_40keV_ to arterial phase images using a 5-Point Likert scale. CI_ven_ images as reference were also available to the readers.

**Table 1:** Likert scores for qualitative assessment

*All patients*
Degree of removal of iodine (contrast media) in virtual non-contrast images derived from venous phase images as compared the true non-contrast images
(5) complete removal of contrast media
(4) almost complete removal of contrast media
(3) moderate removal of contrast with little incomplete areas
(2) partly sufficient removal of contrast media with incomplete areas
(1) insufficient removal of contrast media
Enhancement of the abdominal/pelvic arteries in VMI_40keV_ of venous phase as compared to arterial phase
(5) identical contrast
(4) slightly reduced contrast
(3) moderately reduced contrast but still acceptable
(2) severely reduced contrast
(1) insufficient contrast
*Abdominal aortic aneurysm repair*
Assessibility of aneurysm graft (*VMI*_*40keV*_* and VNC vs Arterial phase and TNC)*
(5) excellent, comparable to conventional images
(4) good, almost comparable to conventional images
(3) still acceptable diagnostic quality
(2) significantly hampered assessment
(1) insufficient diagnostic quality
*Kidney donors*
Evaluation of arterial [and venous] vessel anatomy of the kidneys (*VMI*_*40keV*_* and VNC vs Arterial phase and TNC*)
(5) excellent, comparable to conventional images
(4) good, almost comparable to conventional images
(3) still acceptable diagnostic quality
(2) significantly hampered assessment
(1) insufficient diagnostic quality

### Statistical methods

Continuous variables are reported as mean ± standard deviation (SD). Shapiro–Wilk test revealed non-normal distribution of quantitative and qualitative data. Accordingly, non-parametric Wilcoxon signed rank test was used to account for differences between VNC/TNC and VMI_40keV_/arterial phase images values using JMP software (Version 13, SAS Institute, Cary, USA). Signal-to-noise (SNR) and contrast-to-noise ratio (CNR) were calculated as follows:

$${\text{SNR}} = \frac{{{\text{HU}}_{{\text{vessel lumen}}} }}{{{\text{SD}}_{{\text{vessel lumen}}} }}\;{\text{and}}\;{\text{CNR}} = \frac{{\left| {{\text{HU}}_{{{\text{vessel}}\,{\text{lumen}}}} - {\text{HU}}_{{{\text{muscle}}}} } \right|}}{{\sqrt[2]{{\left( {{\text{SD}}_{{{\text{vessel}}\,{\text{lumen}}}} } \right)^{2} + \left( {{\text{SD}}_{{{\text{muscle}}}} } \right)^{2} }}}}.$$

## Results

### Patients

Of the 25 patients included, 13 were men and 12 were women and the mean age was 54.8 ± 16.6 years. Mean CTDIvol was 11.26 ± 4.0 mGy for TNC examinations, 11.3 ± 4.0 mGy for arterial phase acquisitions, and 11.26 ± 4.0 mGy for venous phase acquisitions, resulting in an average dose of 22.5 ± 8.01 mGy for biphasic scans (i.e., arterial and venous phase) and 33.3 ± 11.8 mGy for triphasic scans (i.e., TNC, arterial and venous phase) which were both significantly higher as the mean effective dose encountered for the monophasic, portal-venous phase scans alone (*p* < 0.05). The corresponding potential dose savings were 11.26 mGy or 50% and 22.5 mGy or 66.7%, respectively.

### Quantitative assessment

**Fig. 1:**
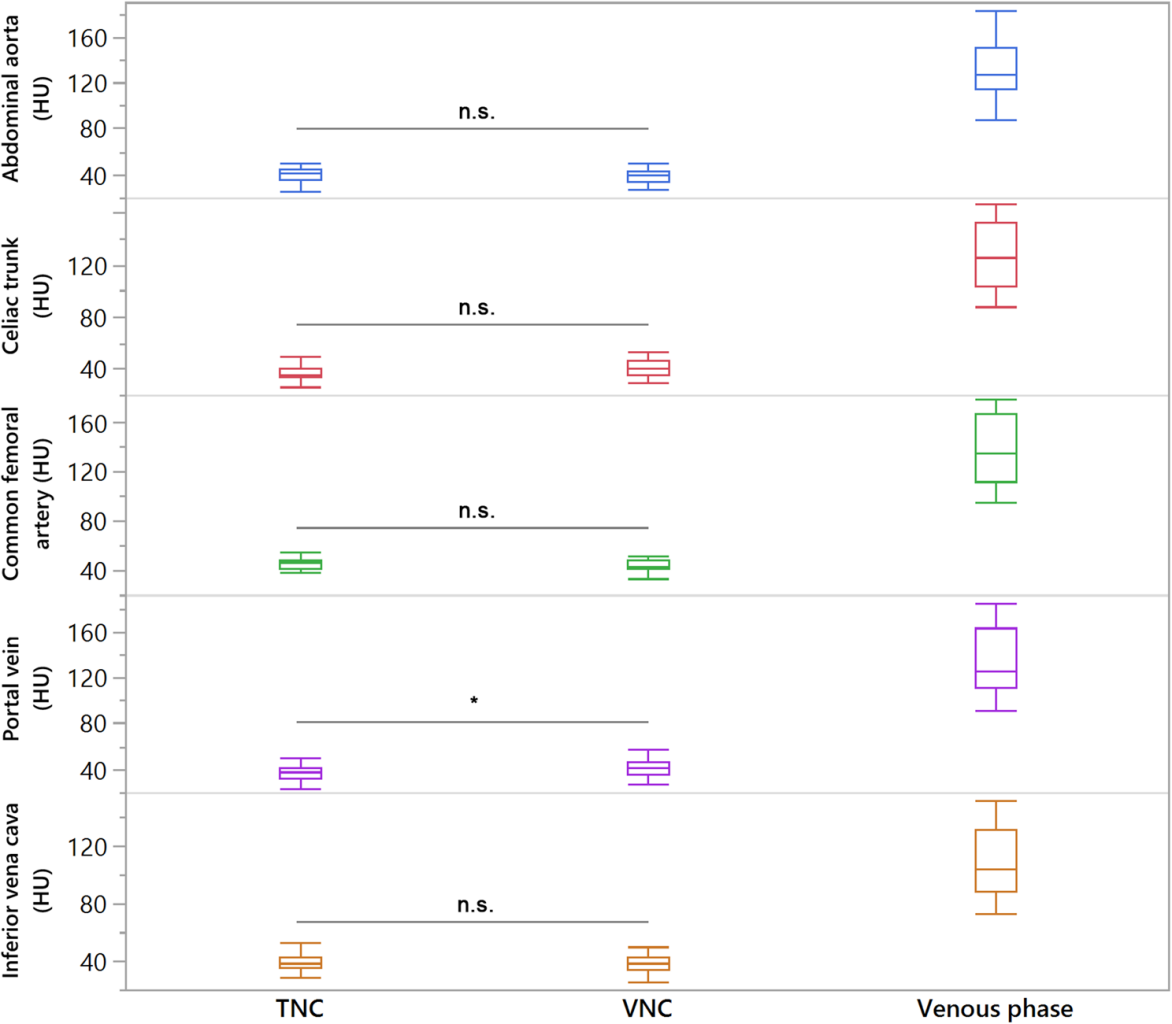
Box-plots for attenuation measurements in true non-contrast (TNC), virtual non-contrast (VNC), and venous phase images showing equivalence for abdominal aorta, celiac trunk, common femoral artery, portal vein, and inferior vena cava. n.s. indicating no significant difference, Asterisks indicating a significant difference

No significant differences in attenuation were found between TNC and VNC images for the abdominal aorta, the celiac trunk, the SMA, the common iliac, external and internal iliac arteries and the common femoral artery (*p*-range 0.070–0.469; Fig. 1), while in the renal artery, VNC attenuation was significantly higher than in TNC (43.4 ± 9.4* HU vs 37.8 ± 9.0, *p* < 0.05).

Attenuation in the inferior vena cava (39.6 ± 5.6 vs. 38.6 ± 6.7, *p* = 0.261) was comparable between TNC and VNC, whereas in the common iliac vein (43.1 ± 6.1 vs. 35.6 ± 6.5, *p* < 0.05) and common femoral vein (43.5 ± 7.9 vs. 38.7 ± 6.1, *p* < 0.05) attenuation was higher in TNC compared to VNC, while in the renal (36.0 ± 8.0 vs. 41.4 ± 6.2, *p* < 0.05) and portal vein (36.9 ± 5.8 vs. 41.1 ± 6.7, *p* < 0.05), it was higher in VNC. For veins in which significant differences between TNC and VNC were found, they were within a range of 10 HU in most patients (portal vein: 22/25, renal vein: 17/25, common iliac vein: 18/25, and common femoral vein: 19/25 patients). Table 2 provides detailed results on TNC/VNC comparison.

**Table 2:** Comparison of true (TNC) and virtual non-contrast (VNC) images (from delayed venous phase acquisition)

Attenuation (HU)				*p*-value (TNC vs. VNC)
	TNC	VNC	Venous phase	
Abdominal aorta	40.7 ± 6.0	39.1 ± 6.5	132.2 ± 25.4	0.297
Celiac trunk	37.7 ± 6.6	40.9 ± 6.8	126.6 ± 24.7	0.070
SMA	39.4 ± 7.8	43.2 ± 11.6	127.1 ± 26.1	0.117
Renal artery	37.8 ± 9.0*	43.4 ± 9.4*	120.4 ± 23.4	0.011
Common iliac artery	44.7 ± 7.5	44.6 ± 9.4	141.6 ± 33.7	0.385
External iliac artery	45 ± 8.3	46.1 ± 7.9	135.6 ± 23.9	0.287
Internal iliac artery	46.3 ± 7.8	49.9 ± 33.2	145.9 ± 88.4	0.468
Common femoral artery	45.3 ± 5.7	43.7 ± 5.9	137.2 ± 28.1	0.469
Vena cava inferior	39.6 ± 5.6	38.6 ± 6.7	108.3 ± 22.8	0.261
Portal vein	36.9 ± 5.8*	41.1 ± 6.7*	136.3 ± 28.9	< 0.001
Renal vein	36.0 ± 8.0*	41.4 ± 6.2*	124.5 ± 28.5	< 0.001
Common iliac vein	43.1 ± 6.1*	35.6 ± 6.5*	98.9 ± 24.3	< 0.001
Common femoral vein	43.5 ± 7.9*	38.7 ± 6.1*	94.0 ± 28.1	0.012

Asterisks indicate statistically significant differences in attenuation between VNC and TNC images for the corresponding vessel

With regard to the comparison of arterial phase images and VMI_40keV_, no significant differences were found for attenuation in all evaluated arterial vessels (*p*-range 0.055–0.864). Pertaining to venous vessels, VMI_40keV_ showed significantly higher attenuation than arterial phase images for the portal, renal, common iliac and femoral veins (all *p* < 0.05). Detailed results of attenuation measurements are listed in Table 3.

**Table 3:** Comparison of vascular attenuation between VMI_40keV_ (from delayed venous phase) vs. arterial phase imaging

Attenuation (HU)				*p*-value (arterial phase vs. VMI_40keV_)
	Arterial phase	VMI_40keV_	Venous phase	
Abdominal aorta	338.8 ± 74.0	369.1 ± 90.1	132.2 ± 25.4	0.055
Celiac trunk	328.8 ± 71.5	341.2 ± 77.5	126.6 ± 24.7	0.392
SMA	328.6 ± 70.2	332.3 ± 83.4	127.1 ± 26.1	0.704
Renal artery	306.5 ± 74.3	309.3 ± 78.2	120.4 ± 23.4	0.864
Common iliac artery	336.3 ± 83.2	375.4 ± 106.7	141.6 ± 33.7	0.283
External iliac artery	313.1 ± 77.0	343.6 ± 74.1	135.6 ± 23.9	0.077
Internal iliac artery	291.7 ± 118	367.4 ± 184.5	145.9 ± 88.4	0.140
Common femoral artery	315.6 ± 102.7	346.0 ± 84.2	137.2 ± 28.1	0.378
Vena cava inferior	60.0 ± 22.7*	281.2 ± 78.0*	108.3 ± 22.8	< 0.001
Portal vein	70.0 ± 24.5*	387.1 ± 103.2*	136.3 ± 28.9	< 0.001
Renal vein	107.3 ± 44.6*	334.4 ± 95.3*	124.5 ± 28.5	< 0.001
Common iliac vein	46.9 ± 8.0*	247.8 ± 79.8*	98.9 ± 24.3	< 0.001
Common femoral vein	44.6 ± 12.9*	210.3 ± 91.3*	94.0 ± 28.1	< 0.001

Asterisks indicate statistically significant differences in attenuation found between arterial phase images and VMI_40keV_ for the corresponding vessel

**Fig. 2:**
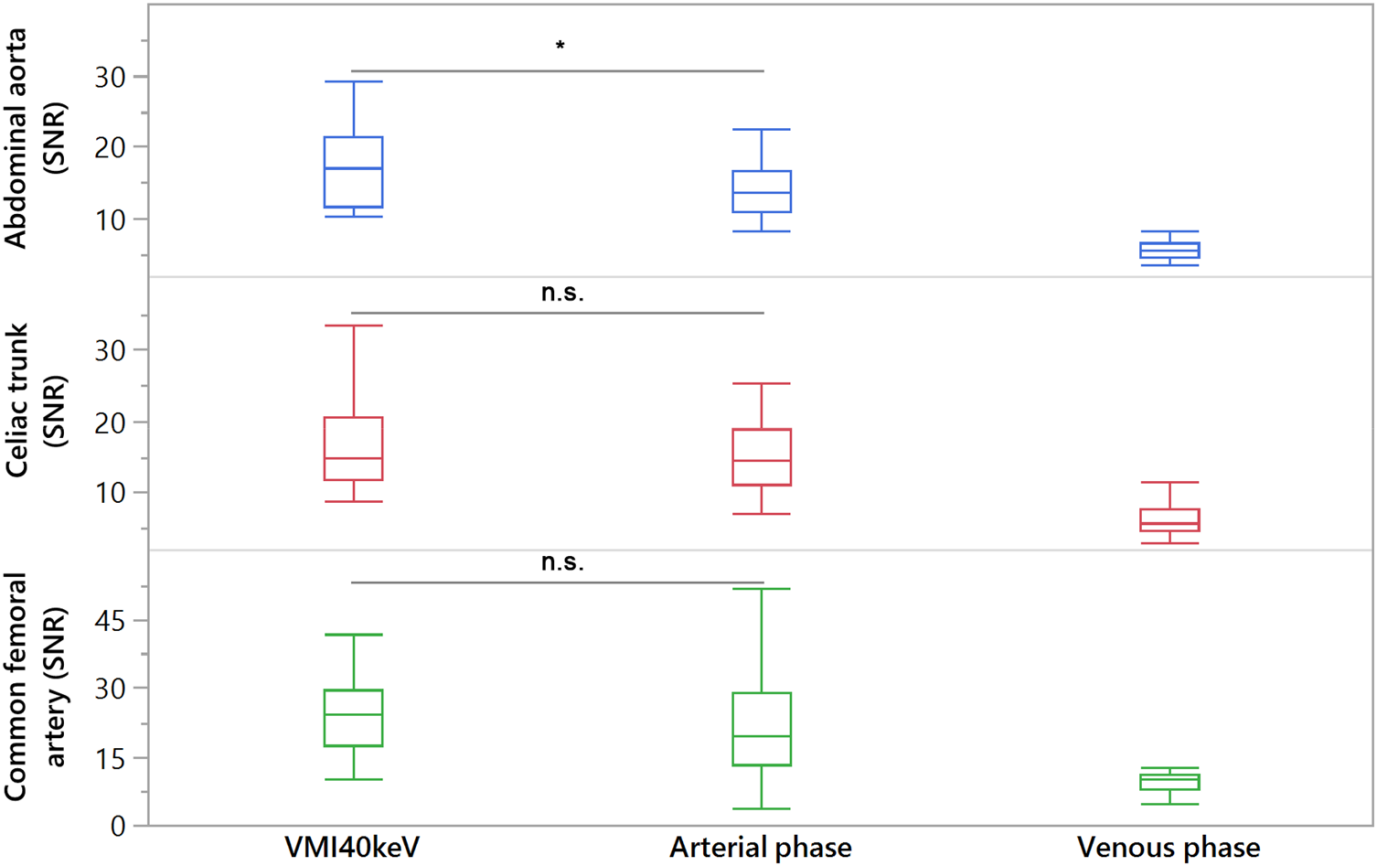
Boxplots demonstrating Signal-to-noise ratios of virtual monoenergetic images at 40 keV (VMI) compared to arterial phase and venous phase images focusing on abdominal aorta, celiac trunk, and common femoral artery. n.s. indicating no significant difference, Asterisks indicating a significant difference

**Fig. 3:**
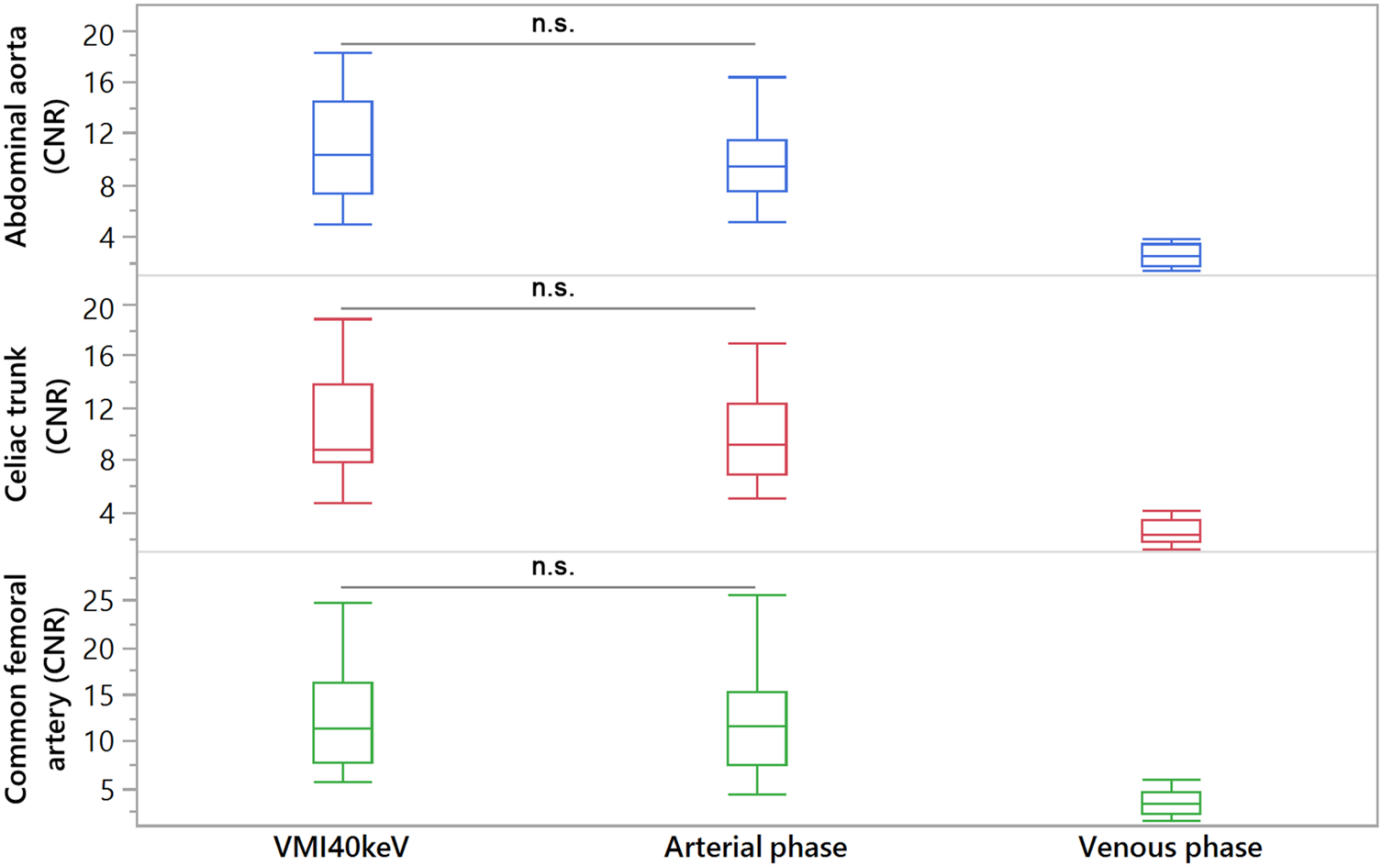
Boxplots demonstrating Contrast-to-noise ratios of virtual monoenergetic images at 40 keV (VMI) compared to arterial phase and venous phase images focusing on abdominal aorta, celiac trunk, and common femoral artery. n.s. indicating no significant difference

SNR was comparable between VMI_40keV_ and arterial phase images for the celiac trunk, SMA, renal artery and common femoral artery (*p*-range 0.162–0.470; Table 4 and Fig. 2), while it was significantly higher in VMI_40keV_ for the abdominal aorta as well as the common, internal and external iliac arteries (*p* < 0.05). CNR was equivalent between VMI_40keV_ and arterial phase images for all evaluated arterial vessels (*p*-range 0.140–0.906; Table 5 and Fig. 3).

**Table 4:** Quantitative comparison of Signal-to-noise ratio between VMI_40keV_ (from venous phase) vs. arterial phase acquisition

Signal-to-noise ratio				*p*-value (arterial phase vs. VMI_40keV_)
	Arterial phase	VMI_40 keV_	Venous phase	
Abdominal aorta	14.7 ± 4.9*	17.4 ± 5.6*	5.6 ± 1.3	0.042
Celiac trunk	15.1 ± 4.9	16.7 ± 6.0	6.4 ± 2.3	0.217
SMA	15.5 ± 6.0	17.8 ± 8.0	6.7 ± 2.7	0.162
Renal artery	13.9 ± 5.4	15.3 ± 6.3	5.8 ± 1.9	0.470
Common iliac artery	15.0 ± 6.6*	19.3 ± 7.7*	6.9 ± 2.2	0.015
External iliac artery	14.5 ± 5.7*	17.5 ± 5.4*	7.0 ± 2.0	0.046
Internal iliac artery	12.1 ± 7.3*	17.0 ± 8.9*	6.6 ± 2.8	< 0.001
Common femoral artery	22.4 ± 12.3	25.7 ± 12.3	9.8 ± 2.9	0.217

Asterisks indicate statistically significant differences between signal-to-noise ratio in arterial phase images and VMI_40keV_

**Table 5:** Quantitative comparison of Contrast-to-noise ratio between VMI_40keV_ (from venous phase) vs arterial phase acquisition

Contrast-to-noise ratio				*p*-value (arterial phase vs. VMI_40keV_)
	Arterial phase	VMI_40 keV_	Venous phase	
Abdominal aorta	10.0 ± 3.5	10.8 ± 3.7	2.6 ± 0.8	0.407
Celiac trunk	9.7 ± 3.2	10.0 ± 3.7	2.5 ± 1.0	0.906
SMA	10.0 ± 3.7	10.0 ± 4.3	2.7 ± 1.2	0.885
Renal artery	8.8 ± 3.5	8.9 ± 3.6	2.3 ± 0.9	0.885
Common iliac artery	9.9 ± 4.5	10.9 ± 4.3	2.9 ± 1.0	0.249
External iliac artery	9.1 ± 3.5	10.1 ± 3.2	2.9 ± 1.0	0.559
Internal iliac artery	7.9 ± 5.1	9.4 ± 4.3	2.7 ± 1.2	0.140
Common femoral artery	11.7 ± 5.9	12.3 ± 4.7	3.5 ± 1.3	0.666

### Qualitative assessment

For qualitative assessment of removal of contrast media in VNC, readers indicated complete contrast media removal and equivalence to TNC images (5 score on Likert Scale) in 90% of cases for the abdominal aorta, in 94% for aortic branches, 88% for pelvic arteries and 100% for abdominopelvic veins.

Enhancement of abdominal and pelvic arteries in VMI_40keV_ from venous-phase images was rated as identical to arterial-phase images (5 score on Likert Scale) for the abdominal aorta, its direct branches and pelvic arteries in 84%, 50%, 78% of the cases, respectively.

In the subgroup of patients with abdominal aortic graft repairs, the graft was evaluated using a combination of VNC and VMI_40keV_ images derived from venous phase. Compared to a combination of TNC and arterial phase images, VNC and VMI_40keV_ were rated equivalent in 85% of the cases.

**Fig. 4:**
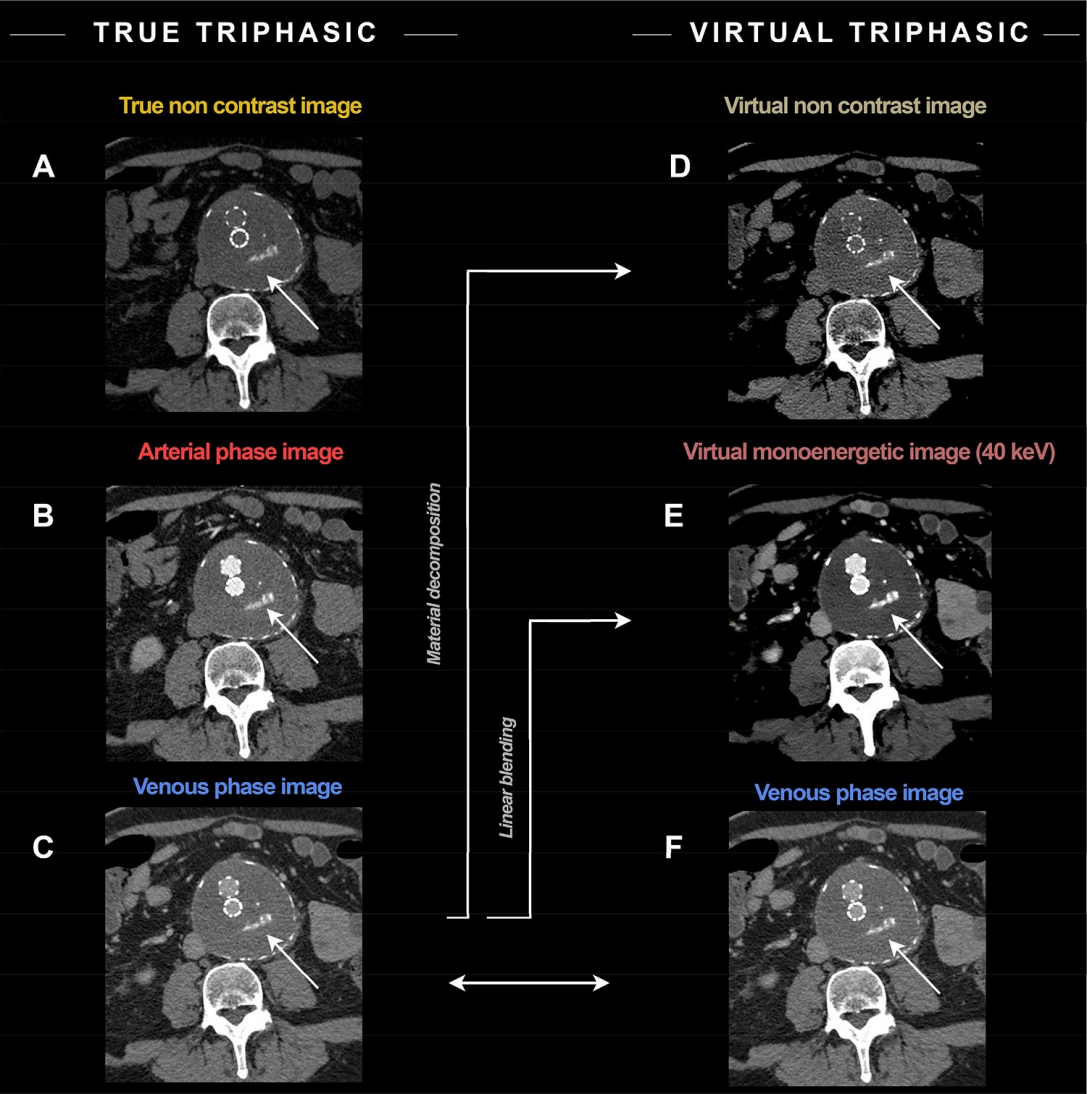
A 64-year-old male with history of abdominal aortic aneurysm repair underwent a follow-up triphasic SDCT examination of the abdomen and pelvis. Axial true non-contrast (TNC) image (**a**) demonstrates areas of hyperattenuation outside the stent/graft but within the lumen of the excluded abdominal aortic aneurysm (white arrows), which is similar in size and configuration to arterial phase image (**b**) and does not change on venous phase image (**c**). The findings are consistent with calcification in the excluded abdominal aortic aneurysm and not an endoleak. When utilizing the virtual non-contrast image (**d**) and virtual monoenergetic image at 40 keV (**e**), derived from the single venous phase, comparable information to triphasic exam can be obtained. Window levels were adjusted for visualization purposes

**Fig. 5:**
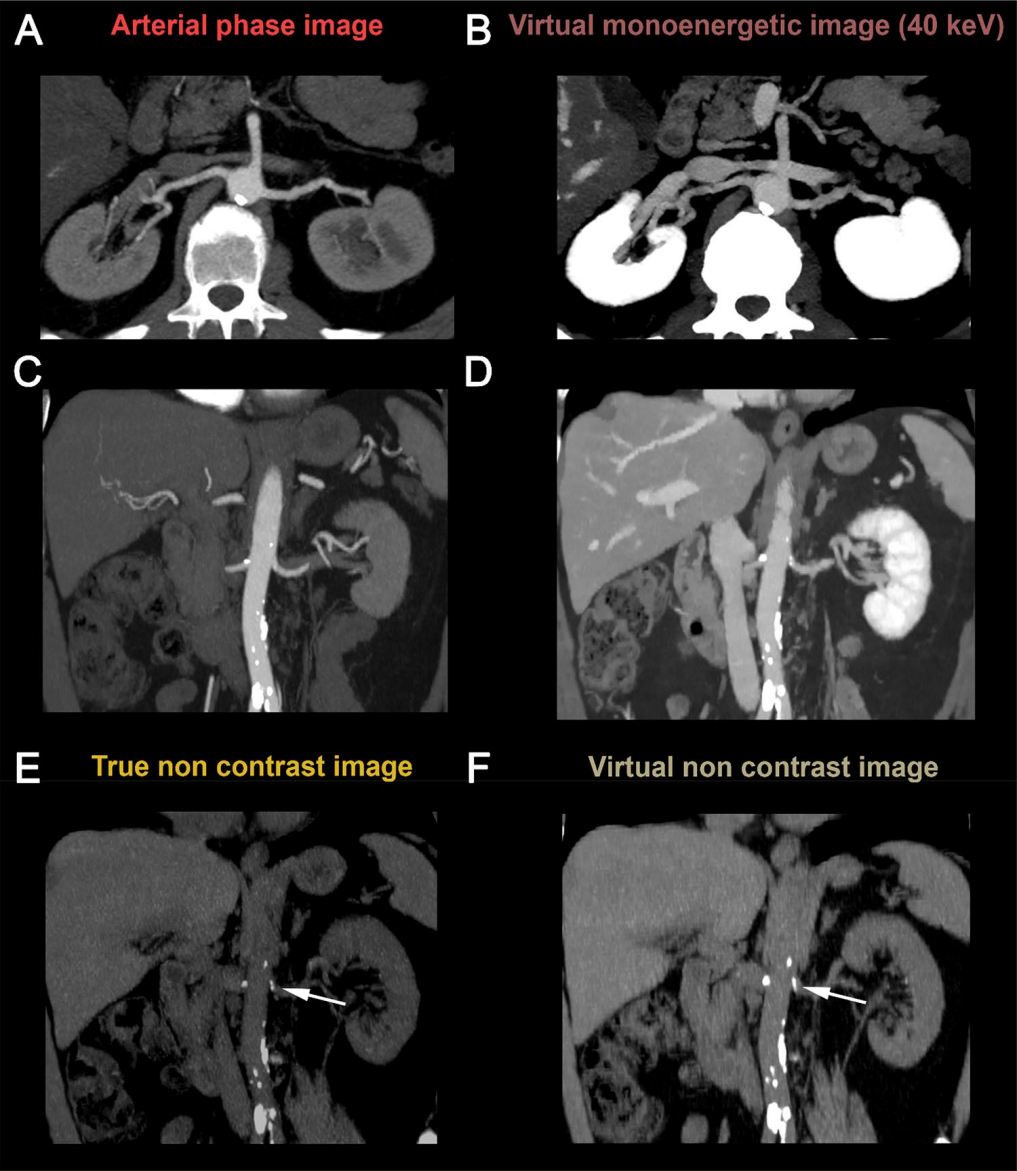
A 55-year-old female underwent a triphasic SDCT examination as part of kidney donor evaluation. Initial arterial phase images in axial and coronal planes (**a**, **c**) demonstrate single renal artery on both sides. The true non-contrast image (**e**) reveals aortobiliac atherosclerosis and bilateral renal ostial calcifications (white arrow). 40 keV images from the delayed phase scan in axial and coronal planes (**b**, **d**) provide strong boost to contrast enhancement of the abdominal aorta and the renal arteries comparable to arterial phase scan (**a**, **c**). VNC images (**f**) allow comparable assessment of atherosclerotic burden (white arrows). Window levels were adjusted for visualization purposes

In the subgroup of patients with kidney donor protocol the depiction of the arterial and venous vessel anatomy of the kidneys was considered fully equivalent in only 15% of cases. However, the depiction of the vasculature was considered acceptable or better in 95% of the cases. Table 6 shows detailed results of the qualitative assessment for each of the two readers. Figures 4 and 5 depict exemplary cases of patients with abdominal aortic aneurysm repair and kidney donor evaluation, respectively.

**Table 6:** Qualitative comparison of VNC and VMI_40keV_ derived from venous-phase images to TNC and arterial phase images by two expert readers

	5	4	3	2	1
*Reader 1*					
Removal of contrast media information					
Abdominal aorta	24/25 (96%)	1/25 (4%)	0/25 (0%)	0/25 (0%)	0/25 (0%)
Direct aortic branches	24/25 (96%)	0/25 (0%)	0/25 (0%)	0/25 (0%)	0/25 (0%)
Pelvic arteries	24/25 (96%)	1/25 (4%)	0/25 (0%)	0/25 (0%)	0/25 (0%)
Abdominopelvic veins	25/25 (100%)	0/25 (0%)	0/25 (0%)	0/25 (0%)	0/25 (0%)
Contrast of the abdominal/pelvic arteries in VMI_40keV_ compared to arterial phase					
Abdominal aorta	20/25 (80%)	4/25 (16%)	1/25 (4%)	0/25 (0%)	0/25 (0%)
Direct aortic branches	14/25 (56%)	7/25 (28%)	4/25 (16%)	0/25 (0%)	0/25 (0%)
Pelvic arteries	21/25 (84%)	4/25 (16%)	0/25 (0%)	0/25 (0%)	0/25 (0%)
Assessability of aneurysm graft					
	7/7 (100%)	0/7 (0%)	0/7 (0%)	0/7 (0%)	0/7 (0%)
Evaluation of arterial [and venous] anatomy of the kidneys					
	3/10 (30%)	5/10 (50%)	2/10 (20%)	0/10 (0%)	0/10 (0%)
*Reader 2*					
Removal of contrast media information					
Abdominal aorta	21/25 (84%)	4/25 (16%)	0/25 (0%)	0/25 (0%)	0/25 (0%)
Direct aortic branches	23/25 (92%)	2/25 (8%)	0/25 (0%)	0/25 (0%)	0/25 (0%)
Pelvic arteries	20/25 (80%)	5/25 (20%)	0/25 (0%)	0/25 (0%)	0/25 (0%)
Abdominopelvic veins	25/25 (100%)	0/25 (0%)	0/25 (0%)	0/25 (0%)	0/25 (0%)
Contrast of the abdominal/pelvic arteries in VMI_40keV_ compared to arterial phase					
Abdominal aorta	22/25 (88%)	3/25 (12%)	0/25 (0%)	0/25 (0%)	0/25 (0%)
Direct aortic branches	11/25 (44%)	11/25 (44%)	3/25 (12%)	0/25 (0%)	0/25 (0%)
Pelvic arteries	18/25 (72%)	7/25 (28%)	0/25 (0%)	0/25 (0%)	0/25 (0%)
Assessability of aneurysm graft					
	5/7 (71,4%)	2/7 (28,6%)	0/7 (0%)	0/7 (0%)	0/7 (0%)
Evaluation of arterial [and venous] anatomy of the kidneys					
	0/10 (0%)	4/10 (40%)	5/10 (50%)	1/10 (10%)	0/10 (0%)

## Discussion

Majority of routine abdominopelvic CT scans are performed as monophasic examinations, yet acquisition of additional unenhanced and arterial phase images may be required for specific indications such as renal donor evaluation or dedicated vascular assessment (e.g., for evaluation of endoleaks of abdominal aortic aneurysm repair or active bleeding) [6, 16]. Whereas the use of DECT-derived VNC and VMI has mostly been investigated separately [8, 11, 14, 17–19] while the combination of these reconstructions for abdominal vessel assessment in spectral detector CT (SDCT) has not been studied yet.

In our study, we aimed to assess whether VNC and VMI_40keV_ derived from venous-phase images acquired with a spectral-detector CT (SDCT), a detector-based DECT, could provide comparable quantitative image parameters and qualitative assessment to TNC and arterial phase images in patients who underwent triphasic examinations. We also investigated two clinical scenarios for which triphasic scans are routinely obtained at our institution: assessment of grafts after abdominal aortic aneurysm repair and evaluation of kidney donors [6, 20].

We found that VNC images were comparable to TNC images with regards to attenuation values in the arterial vessels. This is in line with current literature demonstrating that VNC from dual-energy CT is capable of contrast media removal and creation of VNC images, although reported accuracies slightly differ between studies [9, 18, 19, 21–24]. In venous vessels, differences between VNC and TNC images were more pronounced. This finding adds to recent studies which elucidated that VNC provide reasonable approximations of TNC, yet might lack the accuracy needed for dedicated threshold-based lesion characterization [25]. However, the differences between VNC and VNC we found were mostly within a 10 HU margin that has previously deemed acceptable for TNC/VNC agreement [21]. Moreover, the lower TNC/VNC agreement in venous vessels was not reflected in the qualitative results. In synopsis, we assume that the clinical impact of this potentially lower agreement on vascular assessment will be limited, yet it requires further investigation.

VMI_40keV_ derived from venous phase acquisitions yielded comparable or higher attenuation, SNR, and CNR compared to arterial phase images. These findings are in line with current literature with recent studies demonstrating the usefulness of low-keV VMI to relevantly increase attenuation in abdominal vessels and raise vascular enhancement comparable to arterial phase acquisitions [8, 17, 26–28]. These findings are supported by the qualitatively assessment, at which readers deemed the image contrast provided by VMI_40keV_ as equivalent or merely slightly reduced compared to arterial phase images in the majority of cases.

Regarding the evaluation of postinterventional abdominal aortic aneurysm repair patients and the evaluation of vascular anatomy in kidney donor patients, our results suggest that the combination of VNC and VMI_40keV_ from single-phase scan might offer a replacement for multiphasic imaging. In younger patients and patients undergoing serial imaging, this might be an alternative to reduce radiation dose by almost two thirds, but it needs to be considered that our subgroups were very small. Due to this reason readers could only evaluate assessability of SDCT reconstructions compared to conventional images.

We acknowledge that our retrospective study has some limitations. First, the patient cohort is small, particularly in the individual patient subgroups where triphasic SDCT was performed for abdominal aortic aneurysm repair assessment or presurgical planning of kidney donation. While we consider our results as initial and preliminary, they still suggest that “virtual triphasic” technique may be acceptable for assessment of abdominal aortic aneurysm repair. Nonetheless, this must be verified in large-scale, prospective studies before routine clinical implementation. Second, true blinding regarding VNC and TNC and VMI_40keV_ and arterial phase images was not possible due to the intrinsic characteristic image impression of VNC and VMI_40keV_ reconstructions. We therefore chose a side-by-side approach for the qualitative assessment, accepting the resulting inherent bias. Third, the patients in our study were only scanned on one approach to DECT, i.e., SDCT; however, previous studies have suggested that other DECT systems might show similar advantages. Fourth, although contrast in venous phase could be improved and image quality parameters were comparable to arterial phase images, venous vessels also increased in contrast and might overlap or obscure arterial vessels. Lastly, for some clinical indications, such as liver, renal and adrenal lesions, obtaining dynamic contrast information might be important for definitive characterization. Naturally, this information is lost the proposed virtual triphasic imaging approach.

To conclude, this study showed that VNC and VMI_40keV_ calculated from SDCT-derived venous phase images provide comparable vessel assessment as compared to TNC and arterial phase images, with the caveat of a certain quantitative disagreement between VNC and TNC images in venous vessels. Clinical applications for indications such as abdominal aortic aneurysm repair assessment or kidney donor evaluation should be subject to larger-scale studies verifying our initial results.
